# High throughput cardiac imaging in awake young children: Tips and Tricks

**DOI:** 10.1186/1532-429X-17-S1-T13

**Published:** 2015-02-03

**Authors:** Wendy Norman, Rod Jones, Jennifer Steeden, Vivek Muthurangu

**Affiliations:** 1 Centre of Cardiovascular Imaging, University College London (UCL), London, UK; 2 Cardiac MRI, Great Ormond Street Hospital, London, UK

## Background

Cardiovascular Magnetic Resonance (CMR) Imaging has enabled significant advances in the management of Paediatric Heart disease. However, conventional CMR has three main problems when applied to children. Firstly, the MR environment can be very intimidating for small children. Secondly, many sequences require multiple breath holds, which can be difficult in young or unwell children. Finally, CMR exams can be quite time consuming (upto 1 hour) and this make them difficult to tolerate in the paediatric population. These problems have limited the development of high throughput paediatric CMR. In fact, children under 8 often require general anaesthetic or sedation.

In this study, we describe how combining new advances in CMR technology with simple child friendly scanner policies can make high throughput non-sedated CMR a possibility in children.

## Methods

The rapid imaging protocol described was developed on children with pulmonary hypertension (PH), which is a group of children that require multiple repeat scans in whom general anaesthesia or sedation is relatively contra indicated for diagnostic investigations.

To dispense with breath holds, we use rapid real time imaging techniques. Using radial k-t SENSE, a whole short axis stack with high spatiotemporal resolution (40ms) can be acquired in less than 20 seconds. This allows for validated functional analysis of both ventricles. Flow data can also be obtained using real-time techniques or during free breathing with multiple signal averages.

If the child is small, the parent can lie with the child on the table in the scanner throughout the length of the examination, alleviating fear and providing reassurance. In older children, the parent sits at the end of the scanner where they can see them through a mirror system. Parents are encouraged to lean into the scanner if needed where they can gently stroke their child's head for reassurance. Using the mirror system and headphones, the child can also watch a DVD throughout the scan.

Finally, to reduce total scan time protocols are derived that only focus on clinically necessary information.

## Results

In children with PH our protocol revolves around regular assessment of ventricular function, flow and any shunts. This takes approximately 15-20 minutes.

We now routinely image this group of children from the age of 3 years, on occasions, even younger.

## Conclusions

Using rapid imaging techniques and some practical tricks scanning is possible on even very young children without the need for sedation or GA. We have shown this in our PH patients who have functional and flow data performed within 15-20 minutes, which is tolerable in most children.

## Funding

N/A.

**Figure 1:**
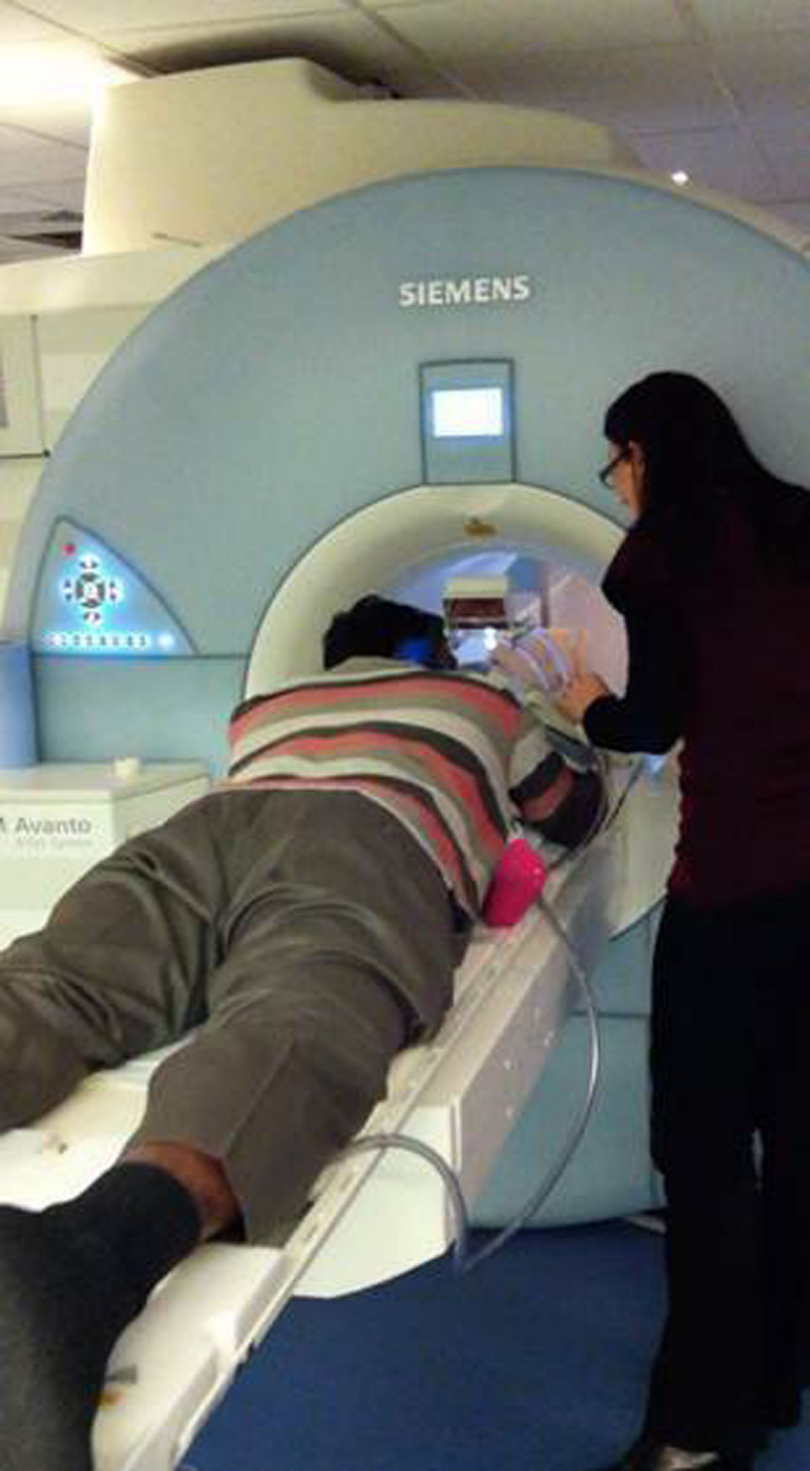
Parent on scanner with child

